# Effects of *Acanthopanax senticosus* on Brain Injury Induced by Simulated Spatial Radiation in Mouse Model Based on Pharmacokinetics and Comparative Proteomics

**DOI:** 10.3390/ijms19010159

**Published:** 2018-01-15

**Authors:** Yingyu Zhou, Cuilin Cheng, Denis Baranenko, Jiaping Wang, Yongzhi Li, Weihong Lu

**Affiliations:** 1 Institute of Extreme Environment Nutrition and Protection, Harbin Institute of Technology, Harbin 150001, China; zhouyingyu_work@outlook.com (Y.Z.); ccuilin@hit.edu.cn (C.C.); 2 National Local Joint Laboratory of Extreme Environmental Nutritional Molecule Synthesis Transformation and Separation, Harbin 150001, China; 3 Biotechnologies of the Third Millennium, ITMO University, Saint-Petersburg 197101, Russia; denis.baranenko@niuitmo.ru; 4 China Astronaut Research and Training Centre, Beijing 100193, China; wangjiaping_1113@163.com

**Keywords:** *Acanthopanax senticosus* (AS), brain injury, pharmacokinetic, proteomics

## Abstract

The active compounds in *Acanthopanax senticosus* (AS) have different pharmacokinetic characteristics in mouse models. C_max_ and AUC of *Acanthopanax senticosus polysaccharides* (ASPS) were significantly reduced in radiation-injured mice, suggesting that the blood flow of mouse was blocked or slowed, due to the pathological state of ischemia and hypoxia, which are caused by radiation. In contrast, the ability of various metabolizing enzymes to inactivate, capacity of biofilm transport decrease, and lessening of renal blood flow accounts for radiation, resulting in the accumulation of syringin and eleutheroside E in the irradiated mouse. Therefore, there were higher pharmacokinetic parameters—AUC, MRT, and t_1/2_ of the two compounds in radiation-injured mouse, when compared with normal mouse. In order to investigate the intrinsic mechanism of AS on radiation injury, AS extract’s protective effects on brain, the main part of mouse that suffered from radiation, were explored. The function of AS extract in repressing expression changes of radiation response proteins in prefrontal cortex (PFC) of mouse brain included tubulin protein family (α-, β-tubulin subunits), dihydropyrimidinase-related protein 2 (CRMP2), γ-actin, 14-3-3 protein family (14-3-3ζ, ε), heat shock protein 90β (HSP90β), and enolase 2. The results demonstrated the AS extract had positive effects on nerve cells’ structure, adhesion, locomotion, fission, and phagocytosis, through regulating various action pathways, such as Hippo, phagosome, PI3K/Akt (phosphatidylinositol 3 kinase/protein kinase B), Neurotrophin, Rap1 (Ras-related protein RAP-1A), gap junction glycolysis/gluconeogenesis, and HIF-1 (Hypoxia-inducible factor 1) signaling pathways to maintain normal mouse neurological activity. All of the results indicated that AS may be a promising alternative medicine for the treatment of radiation injury in mouse brain. It would be tested that whether the bioactive ingredients of AS could be effective through the blood–brain barrier in the future.

## 1. Introduction

Cosmic space radiation can destroy biological macromolecules, such as nucleic acids, lipids, and proteins, causing oxidative stress, cell dysfunction, metabolic disorder, degradation of immune organs, and even triggering malignant cancer [1,2]. Sixty-five percent of human body weight is water, which would produce a lot of free radicals upon stimulation with radiation [3]. Berovic et al., through the quantitative analysis of the process of protein radiation damage, demonstrated that water is the main factor for the destruction of enzymes at room temperature, due to free radical-mediated reactions [4,5]. Therefore, inhibiting free radicals, reducing lipid peroxidation, and improving the survival rate of immune cells to ensure normal physiological function for organisms, are currently major methods to prevent and treat the damage that is caused by radiation.

Currently, the radiation protective drugs mainly include thiamine compounds, heterocyclic compounds, hormones, cytokines, vitamins, and organophosphorus compounds. However, some side effects occur, due to the long-term or excess use of these chemical drugs, such as gastrointestinal discomfort, central nervous system abnormalities, and chronic diseases. When compared with chemical drugs, natural medicines have become increasingly recognized therapeutics in treating radiation injury [6,7]. *Acanthopanax senticosus* (AS) is a well-known traditional Chinese herbal drug that is spread and widely cultivated in North Asia. In recent years, the main active compounds in AS have been identified as saponins, flavonoids, eleutheroside, amino acids, trace elements, polysaccharides, volatile oil, and many more. These bioactive ingredients mentioned above have the function of improving body’s hematopoietic ability, protecting human bone marrow mesenchymal cells, scavenging free radicals, repairing oxidative damage, and inhibiting radiation-corresponding proteins [8]. At the same time, they can inhibit the growth, proliferation, and metastasis of esophageal cancer cells, such as Lewis lung cancer, liver cancer, SGC 7901 gastric cancer, and Eca-109 human esophageal cancer, which may be related to their function in regulating cancer cells’ growth cycle and some apoptosis factors. The biological abundance was shown to have a directly proportional relationship with the dosage of AS [9,10]. The biological activity of these compounds, regarding the treatment of space radiation injury, is primarily reflected in pathway activation, production of beneficial cytokines, protection of nucleic acid, or the prevention of immune deficiency.

Our previous animal experiments found that AS had an effect counter to that of radiation on the ability of learning and memory of mice. Therefore, our research builds on this discovery by exploring further the repair effect of AS on radiation-damaged brain. In this research, a comparative proteome approach was used to explore the radiation sensitivity proteins of mice PFC, and pharmacokinetics was also used to detect metabolism characteristics of AS active ingredients in mice with different body states. The metabolic curve can be used to determine whether AS effective ingredients could be effective through the blood–brain barrier.

Pharmacokinetics is a method that applies dynamic principles and mathematical treatment to give a quantitative description of dynamic laws of drugs’ absorption, distribution, metabolism and excretion process in individuals. Many scholars use pharmacokinetics to study the metabolic situation of AS extract in animal models, and to determine the metabolism characteristics and functions of AS active compounds, according to the pharmacokinetic parameters [11,12]. Unfortunately, these pharmacokinetic studies of AS are mainly confined to the normal body, even though it has been reported that the pharmacokinetic behavior of drugs in the ill body differs [13]. Therefore, it is possible to provide new ideas and data based on a study of AS’s function on treating radiation damage by comparing the metabolic characteristics of effective components of AS extract between radiation-injured mouse and normal mouse [14]. In the applied research, proteomics has become one of the effective methods for studying molecular markers, drug targets, biological development, clinical diagnosis, pathology research, drug screening, new drug development, food testing, and even species identification. In this study, through studying the main functions and metabolic pathways of differential proteins in PFC of mouse brain, with the help of bioinformatics, the effects of AS extract on treating and preventing brain injury caused by radiation would be studied [15,16,17]. The two methods mentioned above could help to explore the intrinsic mechanism of AS extract on treating brain injury, which is caused by radiation, and offer data that would form a basis for the development of new supplementary medicine for treating radiation damage [18].

## 2. Results and Discussion

The main active ingredients of AS extract were determined as *Acanthopanax senticosus polysaccharides* (ASPS), flavones, syringin, and eleutheroside E. The AS extract’s extraction rate was 15.41%. In our research group, the preliminary results that highlight the active compounds in different tissues of AS (crude drug) are shown in Table 1 [19]. Therefore, the AS extract was composed of 30.16% ASPS, 51.69% flavonoids, 4.77% syringin, and 10.34% eleutheroside E.

### 2.1. Pharmacokinetics of Active Compounds of Acanthopanax senticosus (AS) Extract in Mouse Model

The decline of plasma concentration varies with drug’s distribution, metabolism, and excretion rate in mice. With the introduction of a time curve, two-compartment modelling was used to process pharmacokinetic analysis. The mouse body was divided into two rooms, one was the central room with fast blood flow and good permeability, the other room was the surrounding room with slow blood flow and slow metabolism.

#### 2.1.1. Pharmacokinetics of *Acanthopanax senticosus polysaccharides* (ASPS) of AS Extract in Irradiated Mice

In recent years, the understanding of AS’s impact effect on brain tissue is mainly that it is involved in inhibiting oxygen free radicals, curbing the oxidation reaction, preventing NO accumulation, improving the function of the biofilm, and preventing the formation of cerebral thrombosis. In addition, AS has also been shown to regulate the process of mediating the excitement and suppression of the central nervous system, and have a good therapeutic effect on related diseases that are caused by decreased learning and memory ability [9,20,21]. According to the special function of AS on brain repair, it is important to understand the metabolic process of the bioactive ingredients in AS to explore related natural medicine supplements. Therefore, in this research, the metabolic features, in vivo, of the main effective compounds in AS, including ASPS, syringin, and eleutheroside E, have been studied. 

ASPS is the major active ingredient of AS, which plays a protective and reparative role in lots of mouse metabolic systems, including hematopoietic system, immune system, urinary system, central nervous system, and digestive system. The anti-radiation effects of ASPS include enhancing the phagocytosis of immune cells, maintaining hematopoietic function, eliminating toxic and harmful free radicals, preventing the growth inhibition of cells caused by radiation, and prohibiting the infiltration of cancerous cells or inflammatory factors in murine model. 

**Figure 1:**
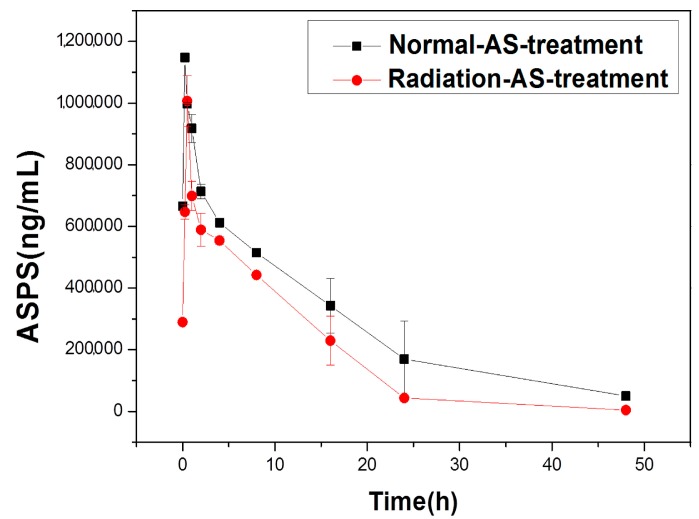
Pharmacokinetics curve of *Acanthopanax senticosus polysaccharide* (ASPS) in mice’s serum.

The metabolic status of ASPS in the normal-AS-treated group and radiation-AS-treated group could be shown in Figure 1, where the pharmacokinetic curves were plotted as a function of time (h) and the content of AS (ng/mL). It was found that the content of ASPS increased, firstly, and then decreased in both the physiological states mentioned above, indicating the process of digestion and metabolism of ASPS in mice. In addition, the ASPS serum concentration for the normal mice and radiation injured mice reached a peak at 0.25 h and 0.5 h, respectively; the former ASPS concentration was always greater than the latter, and the two serum ASPS concentrations in mice had rapid metabolism within 0.5 h–2 h, resulting in its anti-radiation efficacy.

The standard curve of ASPS was *y* = 9.6725*x* − 0.03, *R*^2^ = 0.99958, and the curve had a high degree of fit, which could be used for the following test. With the time (h) as the abscissa, and the concentration of ASPS in serum of mice as the ordinate, the pharmacokinetics curve was obtained, as shown in Figure 1. Then, the PK solver was used to calculate the pharmacokinetic parameters, as shown in Table 2.

The pharmacokinetic curve and parameters of ASPS of AS extract showed significant differences between normal-AS-treated group and radiation-AS treated group as shown in Figure 1 and Table 2. When compared with the normal control group, the time required for elimination of half of the serum drug (t_1/2_; ** *p* < 0.01), and the average residence time (MRT; ** *p* < 0.01) of ASPS were decreased, however, the unit time of drug clearance (CL; * *p* < 0.05) was increased in irradiated mice, indicating that ASPS had a larger demand in sick mice with a higher drug removal speed. Therefore, the results indicate that increasing the number of AS extract deliveries and shortening the time interval of administration would maintain AS’s efficacy on radiation [22]. On the other hand, peak concentration (C_max_) and peak time (T_max_) are two indicators that reflect the rate of drug absorption in vivo. The amount of absorption (AUC; * *p* < 0.05) is an important index to evaluate the degree of drug absorption. From the pharmacokinetic parameters, we could know that the concentration of ASPS in radiation injured mice serum reached a peak at about at 0.5 h, and C_max_ and AUC were significantly reduced in radiation-injured mice, suggesting that blood flow in mice was blocked or slowed by radiation, and that the body transport capacity of mice in the pathogenic state of ischemia and hypoxia was lowered. From Table 2, we could also determine that ASPS had a small apparent distribution volume (V_d_) in the mice model, which illustrated that a few volumes of body fluid were required.

#### 2.1.2. Pharmacokinetics of Syringin and Eleutheroside E of AS Extract in Irradiated Mice

Syringin and eleutheroside E had a much lower concentration in AS, but had an underestimated effect on radiation reparation. Lee et al. found that syringin can regulate biological indicators of oxidation, such as superoxide dismutase (SOD) and glutathione (GSH), removing harmful free radicals, and preventing cell aging and apoptosis [23]. In addition to having the same biological activity as syringin, Huang et al. also found that eleutheroside E had an anti-fatigue effect, which can improve the spatial memory and learning ability of mice [24].

**Figure 2:**
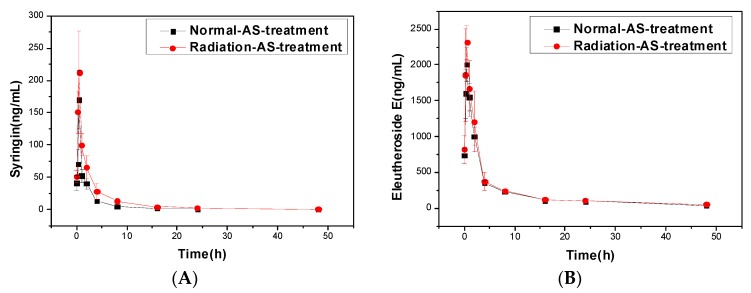
Pharmacokinetics curves of Syringin and Eleutheroside E in mice’s serum. (**A**) Pharmacokinetics curve of Syringin in mice’s serum. (**B**) Pharmacokinetics curve of Eleutheroside E in mice’s serum.

The standard curves of syringin and eleutheroside E were *y* = 9.6725*x* − 0.03, *R*^2^ = 0.9982; and, *y* = 9.6725*x* − 0.03, *R*^2^ = 0.99958, respectively. The two curves had a high degree of fit, which could be used for the following test. The pharmacokinetics curves of the two compounds in serum of mice were shown in Figure 2, and the pharmacokinetic parameters were shown in Table 3.

In contrast, the pharmacokinetic curves and parameters of the other two active substances, syringin and eleutheroside E, had a big difference when compared with ASPS, as shown in Figure 2 and Table 3. From T_max_ and C_max_, we found that the two active substances could have a great effect in regulating physiological activities of irradiated mice in a short time. In order to achieve the active compounds’ distribution and transport, syringin, and eleutheroside E had a higher binding force with plasma proteins in sick mice compared normal mice, which manifested in the decrease of V_d_ (* *p* < 0.05) and CL (** *p* < 0.01), as shown in Table 3. The increase of AUC (** *p* < 0.01) resulted from the accumulation of syringin and eleutheroside E in the mice model. As the drug is mainly metabolized by liver CYP450, the reduced activity of the enzyme under pathological conditions could also reduce the biotransformation of the AS extract. At the same time, in vitro experiments show that syringin and eleutheroside E might be metabolized by intestinal flora [25]. Therefore, we speculated that the decreased ability of various metabolizing enzymes, inactive intestinal flora, and declining biofilm transport were important factors for the change of the pharmacokinetic behavior of the two active compounds in irradiated mice. In addition, the lowering of renal blood flow would reduce the amount of syringin and eleutheroside E of urinary or bile excretion, resulting in their big increase in vivo, and presenting higher MRT (** *p* < 0.01) and t_1/2_ (** *p* < 0.01) in irradiated mice [26]. Additionally, after oral administration of AS extract in mice, syringin, and eleutheroside E may retained in mice intestinal circulation, with the accumulation of AS compounds in the irradiated mice model, which leads to the potential cumulation of poisonous drugs. Therefore, future studies on the disposal process by the body can provide guidance with regards to the clinical safety.

In addition, flavone is also one of the important bioactive compounds in AS. In our preliminary work, only the content of total flavonoids of AS used in the experiment was tested [19]. Since there are a lot of flavonoid compounds in the “total flavonoids” mentioned above, it is difficult to calculate the pharmacokinetic parameters of a specific flavonoid compound. Therefore, we only consider three special compounds in the manuscript, including ASPS, eleutheroside E, and syringin, in radiation-injured mice. This is the second article from our group that relates to the anti-radiation effects of AS. Therefore, the anti-radiation effect of AS extract has been demonstrated from a number of angles, including pharmacokinetics and comparative proteomics. In the future, we will further explore the effects of AS based on the results of the previous experiments. Then, a more specific discussion of the effects of AS on radiation will be studied, including pharmacokinetic characters of the other bioactive compounds in AS, using LC-MS technology.

### 2.2. Effects of AS Extract on Proteins in PFC of Mice Brain after Radiation

**Figure 3:**
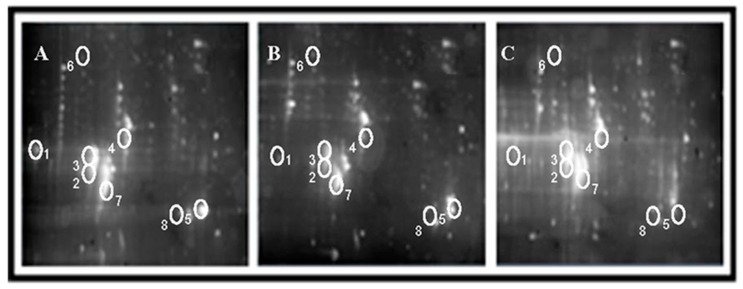
Two-dimensional (2D) gels showing the differences in protein expression and modifications among different groups. (**A**) Normal control group. (**B**) Model set. (**C**) AS extract treatment group. (1. SSP0314; 2. SSP3307; 3. SSP3311; 4. SSP4422; 5. SSP8116; 6. SSP2814; 7. SSP3315; and 8. SSP7119).

Mouse brain proteins were separated by 2-DE. A total of 9 gels (3 individuals × 3 treatment groups) were produced. The protein contents (protein spots numbers in gels) of PFC of each group were as follows: normal control group was 9.54 ± 0.25 mg/mL (260 protein spots), radiation control group was 8.46 ± 0.57 mg/mL (185 protein spots), and AS extract treated group was 8.42 ± 0.29 mg/mL (285 protein spots). There were 22 protein spots that simultaneously satisfied the two conditions, that the gray scale of AS group was more than twice as much as the radiation group and that data had 99% statistical significance. Similarity, there were 18 protein spots that concurrently satisfied the two conditions, that the gray scale of AS group was less than twice as much as the radiation group, and that the data had 99% statistical significance. Eight significantly changed spots among these proteins mentioned above were selected and identified by MALDI mass spectrometry analysis (SSP0314, 3307, 4422, 2814, 3311, 3315, 7119, and 8116), the protein locations and content changes of which are shown in Figure 3 and Table 4.

### 2.3. Results of Bioinformatics Analysis

**Figure 4:**
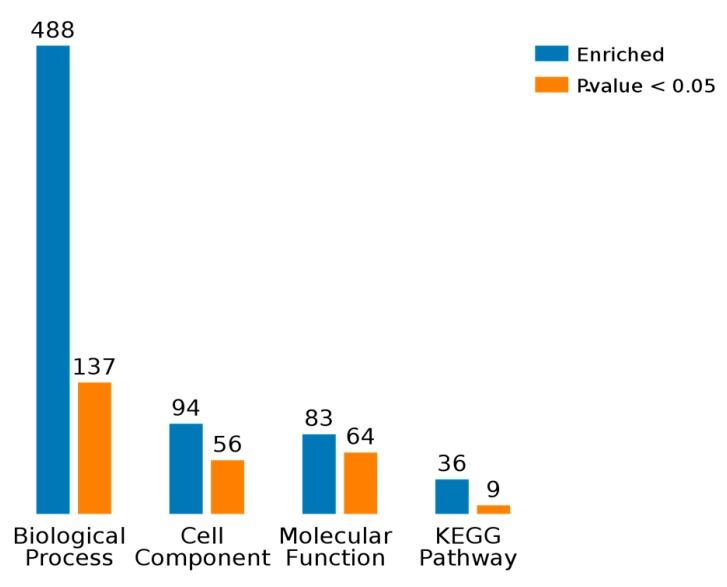
Summary information of each query protein.

As shown in Figure 4, BP, CC, MF, and KEGG are four categories of functions for these eight differentially proteins, and, respectively, correspond to biological process, cellular component, molecule function, and KEGG pathway. Counts for each category represents the total associated terms in the database with the query protein list. Terms with a *p*-value < 0.05 were considered statistically significant. The eight differential proteins were labeled with 488 biological processes, of which 137 were statistically significant. Similarity, 56 of the 94 cell components were statistically significant, 64 of the 83 molecule functions were statistically significant, and 9 of the 36 KEGG pathways were statistically significant.

#### 2.3.1. Gene Ontology Analysis

**Figure 5:**
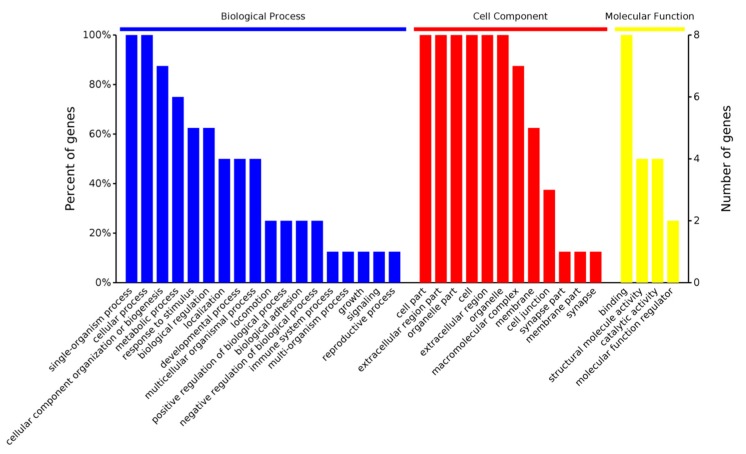
Enriched Gene Ontology.

GO (Gene Ontology) is a major bioinformatics initiative to unify the representation of gene and gene product attributes across all the species. The project aims to maintain and develop its controlled vocabulary, or make annotation of genes and gene products, meanwhile, assimilating and disseminating annotation data. The biological process (BP), cell component (CC), and molecular function (MF) are three basic categories of Gene Ontology. Figure 5 shows that these differentially abundant proteins could take part in various biological process (the blue bars), within many kinds of cell components (the red bars), and achieving multiple molecular functions (the yellow bars).

**Figure 6:**
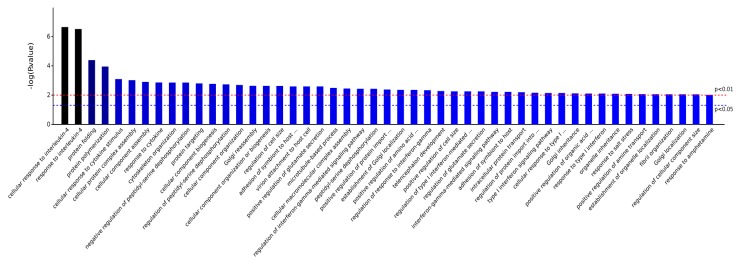
Significantly different Biological Processes.

As shown in Figure 6, the significantly different biological processes involved in the eight proteins included cell composition or biogenesis, bioadhesion, stress responses, reproductive processes, metabolic processes, immune system processes, reactions to chemicals, cell localization, and more. Together, this suggests that the AS extract could regulate various biological functional systems, including the immune system, the reproductive system, metabolic system, etc., thereby, simulating stress responses to prevent the immune cells of mouse brain tissue from external radiation damage.

**Figure 7:**
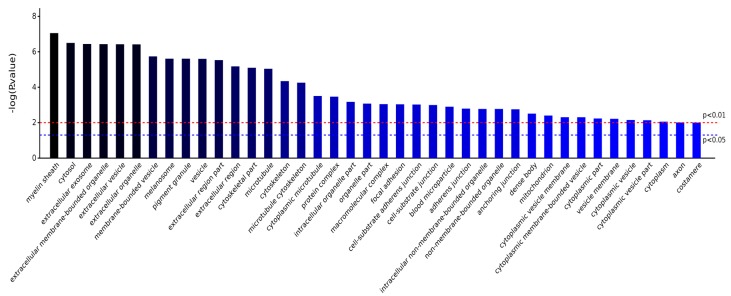
Significantly different Cell Componentes.

As shown in Figure 7, the significantly different cell components that are involved in the eight proteins included myelin sheath, cytosol, extracellular exosome, extracellular membrane-bounded organelle, extracellular organelle, membrane-bounded vesicle, pigment granule, cytoskeleton, protein complex, and intracellular organelle, indicating that AS extract could act on nerve cells’ synaptic structure, thereby promoting signaling and material transport among nerve cells of irradiated mice. In addition, these differentially abundant proteins were related to a variety of biofilm structures, suggesting that AS extract could maintain the stability of mouse brain cells’ metabolism, control substances into and out of brain cells, and play essential roles in cell recognition, cellulase synthesis, or other functions.

**Figure 8:**
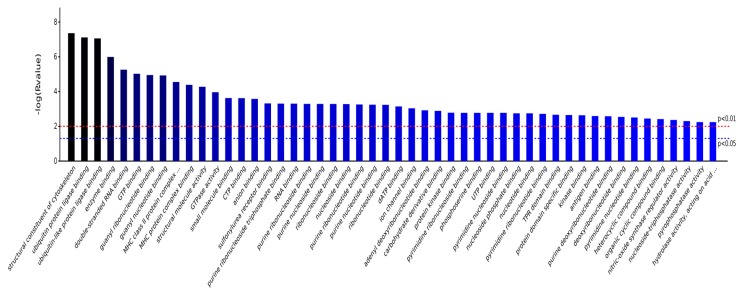
Significantly different Molecular Functiones.

As shown in Figure 8, the significantly different molecule functions that are involved in the eight proteins included structural constituent of cytoskeleton, ubiquitin protein ligase binding, double-stranded RNA binding, GTP binding, guanyl ribonucleotide binding, MHC class II protein complex binding, GTPase activity, CTP binding, anion binding, sulfonylurea receptor binding, purine ribonucleoside triphosphate binding, RNA binding, etc. Ubiquitin is a small molecule protein that is widely present in eukaryotic organisms, which can label proteins that need to be degraded, and then remove them by enzymatic reaction. Ubiquitin, therefore, has the ability to repair nucleic acid damage and regulate the NF-κB pathway. Allof the molecular functions that are involved in the eight differential proteins suggested that AS extracts had reparatory effects on biological macromolecules and the nerve cell cytoskeleton, besides the function of maintaining the normal morphology of cells.

#### 2.3.2. KEGG Pathway Analysis

**Figure 9:**
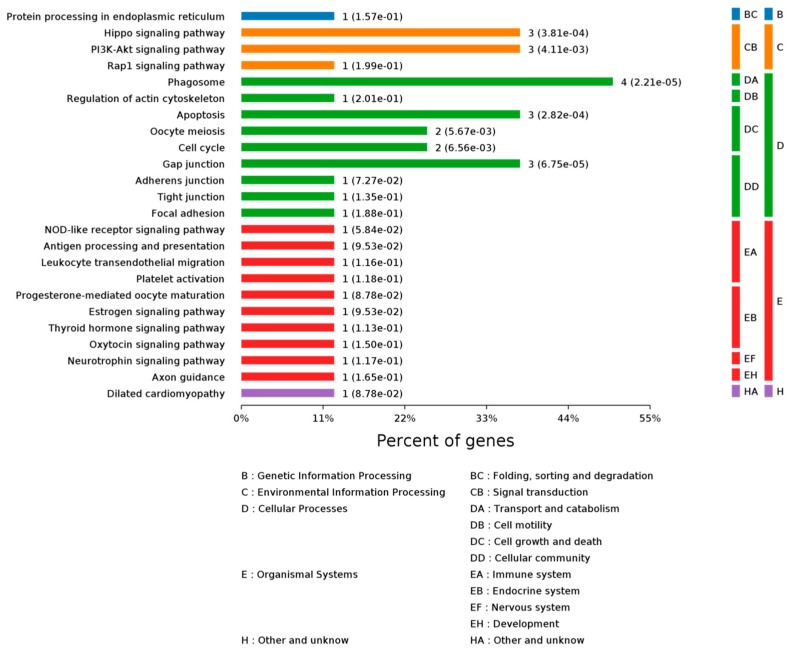
Class of enriched KEGG Pathways.

**Figure 10:**
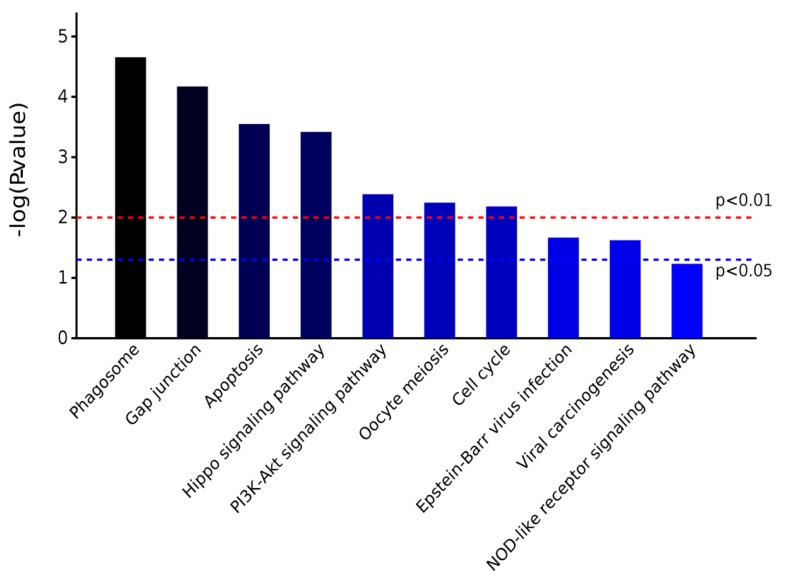
Significantly different KEGG pathways.

KEGG (Kyoto Encyclopedia of Genes and Genomes) is a pathway database. According to KEGG database, pathways are clustered into the following sub-categories, (A) metabolism; (B) genetic information processing; (C) environmental information processing; (D) cellular processes; (E) organismal systems; and, (F) human diseases. Enrichment analysis of KEGG pathways were performed with same hypergeometric algorithm used in gene ontology enrichment analysis. Enriched processes are shown in Figure 9 and Figure 10. There were four differentially abundant proteins that could regulate phagosome signaling pathway, three proteins that could regulate Hippo, P13K/Akt, apoptosis, and Gap junction signaling pathways. The KEGG pathway analysis is shown in Table 5. These results demonstrate the AS extract had a positive function on CNS cells’ structure, adhesion, locomotion, fission, and phagocytosis. Therefore, we demonstrated that AS extract had the ability of maintaining normal neurological activity to prevent brain damage and cancer caused by radiation.

#### 2.3.3. STRING Interactive Network Analysis

**Figure 11:**
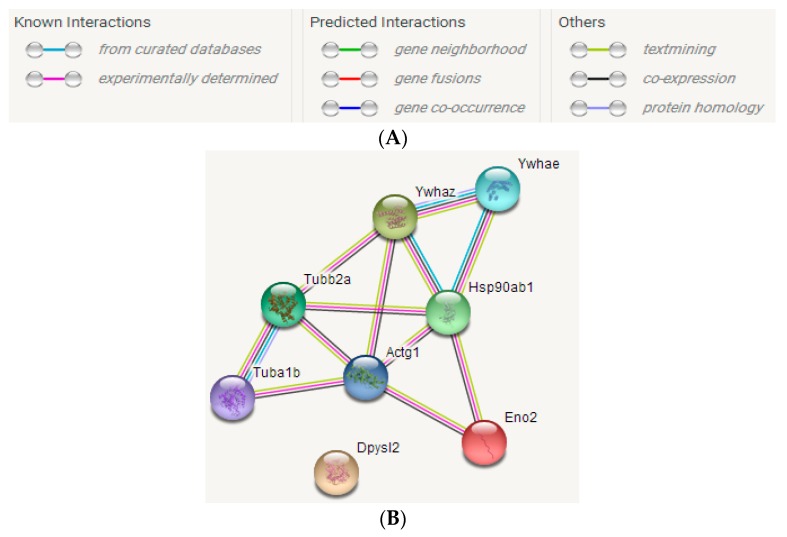
Interactions among differential proteins (**A**) Interaction diagram (**B**) Interaction network among differential proteins.

The interaction relationships among these differentially abundant proteins were discussed to study the repair impacts of AS extract on brain injury caused by radiation, using the STRING website. As shown in Figure 11, the differentially proteins whose gene names were Tuba1b, Tubb2a, Actg1, Ywhaz, Ywhae, Hsp90ab, and Eno2, had strong interaction relationships with each other. The line in different colors between two proteins expressed different interaction relationships, including known interactions (from curated databases and experimentally determined), predicted interactions (gene neighborhood, gene fusions and gene co-occurrence), and others (text mining, co-expression, and protein homology).

### 2.4. Analysis of Differentially Abundant Proteins

#### 2.4.1. Tubulin Protein Family

α-, β-Tubulin proteins are key proteins in neuronal structure and functional recovery in the brain. Tubulin proteins can participate in a variety of cell physiological activities, such as forming cell scaffolds, intracellular transport of substances, maintaining spatial distribution of endometrial organelles, regulating intracellular signal transmission, and cell mitosis modifying processes [27]. The contents of tubulin proteins are associated with various neurological disorders, including Alzheimer’s disease, ischemic brain damage, epilepsy, central nervous system tumors, or depression. From the KEGG pathway analysis (Table 5), the two tubulin proteins are involved in the regulation of apoptosis and phagosome pathways simultaneously. α-Tubulin could also cooperate with actin proteins to regulate thyroid hormone signaling pathway. The proteomic analysis shows that radiation damage caused a decrease in α-tubulin expression, leading to tubulin polymerization and developmental abnormalities, suggesting that the neuronal synaptic function in the central nervous system (CNS) of irradiated mouse was influenced, nerve cell signaling pathways blocked, and that locomotion proteins and axoplasmic flow, which are maintained by α-tubulin, were damaged, and that nerve cells’ normal activities, which depend on synaptic terminals, were affected [28]. The content of β-tubulin protein was also significantly different in PFC of irradiated mouse. β-Tubulin plays an important role in supporting neuronal cytoskeleton and neurotransmitter transport as well. Many reports also see β-tubulin as a prognosis criterion to predict the extent and chemotherapy resistance of malignancy tumors. Radiation injury also has the possibility of poor prognosis in mouse, so, the increase of β-tubulin might be caused by the release of necrotic nerve cells in the blood; the heavier damage in brain, the higher the expression of the protein in the PFC of mouse. However, it could be seen in the experimental analysis that there was not a similar trend in the changes of the two tubulin proteins. The AS extract could promote the production of α-tubulin and inhibit the overexpression of β-tubulin to a certain extent. As some scholars reported, tubulin in the stages of brain injury began to decline after an early increase, followed by the brain producing a stress reaction that resulted in certain proteins’ increasing expression upon injury, followed by a rapid decline, therefore, leading to a variety of neurological diseases [29]. A possible explanation was that the two tubulin proteins had differing regulation cycles of brain radiation injury, and the experiments that assess radiation treatment time as an independent variable, to explore the time-dependent regulatory effects of tubulin on brain function, will be essential in the future.

#### 2.4.2. Dihydropyrimidinase-Related Protein 2 (CRMP2)

CRMP2 plays an important role in axonal growth by regulating the assembly of cell microtubules, endocytosis of adhesion molecules, recombination of actin filaments, and transportation of axonal protein [30,31]. Therefore, CRMP2 has the ability to improve the transmission of signaling molecules among nerve cells. As a substrate for Rho-related proteins, CRMP2 can dynamically regulate of cell microtubules and the process of cytoskeletal remodeling. From the KEGG pathway analysis (Table 5), CRMP2 was involved in regulating the axon-guided pathway, and therefore, played an essential role in axon elongation and neuronal polarization formation. Several articles indicate that the CRMP2 content is positively correlated with the severity of brain damage [32,33]. In this study, we found that the expression of the protein in the model set increased nearly sevenfold, suggesting that the protein is involved in the formation of the PFC neurons’ axons, and the retention of neuronal polarity during the progress of mouse brain injury caused by radiation, at the same time, repairing damaged neurons, forming neural networks, and improving post-oxidized protein conformation. In addition, recent studies have shown that CRMP2 is present in the immune system, playing an especially import role in T lymphocyte function. In peripheral blood, and mononuclear cells of patients with central nervous system inflammatory disease, CRMP2 expression is significantly upregulated, thereby promoting transportation and aggregation of chemokine-mediated T cell, also maintaining the function of injured nerve cells [31].

#### 2.4.3. Actin Proteins

Actin proteins are important skeletal proteins of nerve cells. They are the basic units of microfilaments. In addition to providing the necessary structural framework to determine the shape and polarity of nerve cells, actin proteins’ kinetic changes are also closely related to cell secretion, cytoplasmic circulation, and cytoplasmic division. Myosin is a kind of protein that promotes the movement of microfilaments, which is involved in cell phagocytosis, locomotion, fertilization, absorption, and other physiological processes. γ-Actin, dihydropyrimidine-related protein 2, and tubulin are all cytoskeletal proteins, and these proteins were obviously regulated in the AS treated group. The results suggested that AS extract could promote γ-actin to coordinate with other skeletal proteins to reconstruct the cytoskeleton of brain cells, playing a significant role in maintaining normal morphology, adhesion, migration, and signal transportation of brain cells [31].

#### 2.4.4. 14-3-3 Protein Family

14-3-3 proteins are highly conserved multifunctional proteins of eukaryotes, and have a certain role in the physiological functions of brain. They can react with a variety of signal molecules, such as ASK-1, Fkhr-1, Bcl-2, YAP, Tert, PKB, MAPK, P75, and caspase-3 to regulate apoptosis, promote cell division, and maintain normal cell cycle. 14-3-3 proteins are excessively overexpressed in many human brain diseases, including viral encephalitis, cerebral infarction, hypoxic brain damage, multiple malignant sclerosis, CJD (Creutzfeldt–Jakob disease), and PD (Parkinson’s disease) [34,35]. In this study, a larger number of 14-3-3ζ and 14-3-3ε proteins in the model set were expressed compared with the normal control group and AS treated group. Many studies suggested that 14-3-3ζ can interact with ADAM22 (a disintegrin and metalloprotease domain 22) to regulate cell adhesion and infiltration, or to bind JNK (Jun N-terminal kinase) to induce BAD translocation, thereby affecting JNK-mediated apoptosis and exocytosis. For these reasons, adjusting the expression of 14-3-3ζ can improve the treatment effect of TNF-α (tumor necrosis factor-α) and increase sensitivity of cells’ death, which is caused by serum deprivation [36,37]. Another 14-3-3ε protein is thought to be associated with glial cell activation, as cleavage of 14-3-3ε causes release of their associated apoptotic promoters to influence mitochondrial pathways. In addition, 14-3-3ε can specifically regulate the neurotrophic signaling pathway to promote the growth and differentiation of mice brain cells, increase the signal transduction among neurons, and repair neuronal damage caused by radiation. From KEGG pathway analysis (Table 5) that showed that the 14-3-3 protein involved in this experiment can cooperate with actin to regulate the signal molecules of YAP, MAPK, CK1, and catenin, thereby controlling the hippopotamus signal pathway and preventing brain cell carcinogenesis caused by radiation injury. The expression of 14-3-3 proteins in the brain of irradiated mouse were significantly higher than that in the normal group and the administration group, which indicated that the radiation injury induced the stress reaction of mice brain tissue, which manifested as overexpression of 14-3-3 proteins. Therefore, 14-3-3 proteins may become marker proteins for diagnostic and therapeutic targets on brain tissue lesions.

#### 2.4.5. Heat Shock Protein (HSP) Family

HSP90 is an important protein that regulates activity of estrogen receptor, participates in apoptosis, performs protein folding processes, and plays a role in immune response, maintains cell homeostasis, and more [38]. In this article, the content of HSP90 protein in the AS treated group was significantly higher than that in the model set, indicating that AS could regulate the function of the bioremediation process by inducing the production of heat shock protein when the mice were injured by radiation. HSP90 could promote peptide chain refolding, repair denatured protein, improve cell tolerance for radiation stress, suggesting that AS had the capability to activate macrophages, lymphoid and dendritic cells, thus exerting some of its anti-radiation effects [39]. These results offer evidence for studying radiation-related public occupational hazards, which are of great theoretical and practical significance, to elucidate the mechanism of harmful occupational factors, and explore the prevention and control methods for radiation. As shown in KEGG pathway analysis (Table 5), HSP90 protein is involved in regulating P13K/Akt signaling pathway, which has the essential ability of controlling growth and differentiation of nerve cells, inhibiting nerve cell disease, regulating key pathways related to cell apoptosis, and adjusting various biological processes in mouse brain tumor cells. As changes to radiation-induced kinases always occur in the early and middle stages of tumorigenesis, in this study, it was found that AS extract could significantly alleviate the decrease of HSP90β protein that is caused by radiation in mouse brain, compared with the model set, indicating that AS had great significance in treating radiation-induced glioma and other malignant tumors.

#### 2.4.6. Enolase 2

Enolase 2 has neurotrophic and neuroprotective properties in a broad spectrum of central nervous system (CNS) neurons. Binding in a calcium-dependent manner, enolase 2 can culture neocortical neurons and promote cell survival as well. Enolase 2 is a glycolytic enzyme that is present in neuronal cells and its secretory product is easily released because it does not bind to sarcoplasmic protein. When compared with the AS treatment group, the content of enolase 2 in the model set was significantly increased, indicating that mouse brain was exposed to radiation and then released enolase 2. AS extract alleviated this process, suggesting that AS extract had therapeutic action on a series of follow-up disorders after cerebral hypoxia induced by radiation, including accumulation of lactic acid, formation of acidic environment, normal brain cell growth disorders, invasion of cancer cells, neuronal synaptic damage, abnormal nerve signal transduction, etc. In addition, enolase 2 was also the key protein for protection of nerve cells. It is suggested that the AS extract could regulate neurotrophic signaling pathways by controlling the levels of enolase 2 and 14-3-3ε protein to promote the growth and differentiation of mouse brain cells, increase the signal transduction between synapses, and repair neuronal damage caused by radiation. The KEGG pathway analysis (Table 5) showed that enolase 2 mainly regulated HIF-1 and glycolysis/gluconeogenesis signaling pathways. The glycolysis/gluconeogenesis signaling pathways can provide energy in mouse brain that has suffered hypoxia, and generate synthetic precursors of intermediate metabolites, such as amino acids and lipids. This indicates that AS extract could adjust the glycolysis/gluconeogenesis signaling pathways to clear brain oxygen respiration products, regulate aerobic respiration disorders, and promote the production of protein and lipid compounds, preventing mouse brain protein loss and brain cell necrosis. HIF-1, a key transcriptional activator in mammals under hypoxic physiological states, can regulate the expression of a variety of cytokines and growth-related genes. HIF-1 has strong impacts on mice physiological processes, such as immune system, tolerance, metabolism, growth, proliferation, angiogenesis, and more. In this study, HIF-1 signaling pathways regulated by AS extract in mouse brain illustrated that AS had positive effects on improving the energy supply and normal cellular function of mouse brain cells, which had a strong significance on improving mice tolerance of radiation.

## 3. Materials and Methods

### 3.1. Plant Material

*Acanthopanax senticosus* (AS) was collected on the Lesser Khingan Mountain, Heilongjiang, China, in September 2015. The AS was identified by Prof. Yu Jiabin (Herb Identification and Medicinal Plant Teaching and Research Section, Heilongjiang University of Chinese Medicine). The 4-year-old AS was chosen randomly. The root, stem, leaves, and fruit of AS was separated and stored at −80 °C after freeze-drying. A total of 250 g of each of the roots, stems, leaves, and fruits of AS were individually milled and extracted with distilled water at 65 °C for 3 h. The operation was repeated three times, and the decoctions were combined to obtain the mixture. After that step, the mixed decoctions of all of the parts were filtered with gauze and subsequently concentrated in a rotary evacuator to obtain 50 g of AS extract.

### 3.2. Animal Material

A total of 124 6–8 weeks old male *Kun-Ming* mice, weighing 20.0 ± 5 g, were purchased from the Harbin Medical University, Harbin, Heilongjiang, China, they were maintained under conditions of controlled appropriate temperature (23 ± 2 °C) and humidity (50 ± 5%) with a 12 h light/dark cycle. In addition, the mice were allowed at 1 week to adapt to the environment before being used for the experiments. All of the mice were randomly divided into two large groups. GROUP 1:100 mice were randomly divided into two small groups (*n* = 50 per group) for pharmacokinetics. The two groups comprised of: normal-AS treatment group (235.7 mg/kg/day) and radiation-AS-treatment group (235.7 mg/kg/day). GROUP 2: 24 mice were randomly divided into three small groups (*n* = 8 per group) for the proteomics analysis as follows: Normal control group (without any operation), model set (only radiation without any active compounds), AS extract (235.7 mg/kg/day) treatment group (AS extract plus radiation).

For the GROUP 1: Mice from the radiation-AS treatment group, were irradiated by 60 Co-γ ray irradiation with the mean linear energy transfer (LET) value of 62.2 KeV/µm at a dose of 4 Gy and a dose rate of 0.1 Gy/min. Each mouse was placed in a separate plastic container (20 cm × 20 cm × 100 cm) without anesthetization, and the gauze was used to immobilize the mouse body and enable the head to be exposed to radiation. The radiation lasted 30 min. Then, all of the mice were exposed to aqueous solutions of AS extract (oral administration; 0.25 mL) at the above described dosing level (Chinese Pharmacopoeia 2010). After that, using EDTA-K2 blood collection (Nanjing Jiancheng Bioengineering Institute, Nanjing, China) to get the eyeball blood of each groups’ mice at 0, 0.25, 0.5, 1, 2, 4, 8, 16, 24, and 48 h (every time point five mice would be put to death). These blood were placed into 4 °C refrigerator overnight after standing at room temperature for 2 h. Then, the supernatant of eyeball blood were got after 3000 r/min centrifugation for 10 min. The serum was preserved at −20 °C standby.

For the GROUP 2: Mice were exposed to aqueous solutions of the active compounds (AS extract treatment group) daily (oral administration at 8:00 a.m.; 0.25 mL) at the above described dosing levels (Chinese Pharmacopoeia 2010) over a 14 day period. The mice in the normal control group and model set were given normal saline (NS) daily on the basis of equal volume. All mice, except those from the normal control group, were irradiated in the same condition mentioned above. Then, they were euthanized by cervical dislocation at three days after irradiation. Their brains were used for the proteomics analysis.

All of the experimental protocols were approved by the Experimental Animal Ethic Committee of Harbin Medical University, Harbin, China (Animal Experimental Ethical Inspection Protocol No. 2013011, 8 May 2016). The animal procedures used were consistent with the Guide for the Care and Use of Laboratory Animals, which was published by the US National Institutes of Health (NIH Publication No. 85–23, revised 1996).

### 3.3. Multi-Component Pharmacokinetics of AS Extract

#### 3.3.1. Pharmacokinetics of ASPS

*Acanthopanax senticosus polysaccharide* (ASPS) content’s calculation for every time points in mice’s serum were determined by the phenol-sulfuric acid method, using d-glucose as the standard [40]. Briefly, the serum of mice was dissolved in distilled water to obtain an appropriate concentration solution, then phenol and concentrated sulfuric acid were added sequentially. After vortexing and standing for 30 min at room temperature, the absorbance of the solution was measured by spectrophotometer (Shimadzu-UV-2450, Shanghai, China) at 490 nm. ASPS content was expressed as nanograms of d-glucose equivalents per milliliter of mice serum. Then, PKsolver was used to calculate the relevant pharmacokinetic parameters.

#### 3.3.2. Pharmacokinetics of Syringin and Eleutheroside E

The content of syringin and eleutheroside E in the samples for each time points was detected by HPLC. An Agilent 1100 series liquid chromatograph (Agilent Technologies, Santa Clara, CA, USA), equipped with a quaternary gradient pump and a UV detector was used. A HPLC method was developed using a reversed-phase C18 column (Agilent-TC, 250 mm × 4.6 mm, 5 μm i.d.) with the column temperature at 30 °C. Sample injection quantity was 20 μL; the elution solvent consisted of water (A, with 0.1% phosphoric acid) and acetonitrile (B) with the following gradient program: 0–10 min, 90% A; 10–20 min, 85% A; 20–30 min, 80% A; 30–40 min, 75–90% A; and, 40–50 min, 90% A. The flow rate was kept at 1 mL/min, and the absorbance was measured at a wavelength of 220 nm for Syringin, 207 nm for eleutheroside E [41,42]. The retention times are 6.041 min and 8.500 min for Syringin and eleutheroside E respectively. Contents of syringin and eleutheroside E were expressed in each case as nanograms of syringin and eleutheroside E per milliliter of mice serum. Then, PKsolver was used to calculate the relevant pharmacokinetic parameters.

### 3.4. Two-Dimensional Gel Electrophoresis (2-DE) Analysis

#### 3.4.1. 2-DE of Prefrontal Cortex (PFC)

The mice brains were separated on an ice plate with a temperature of −70 °C. The brain tissues were rinsed with pre-cooled three-distilled water (Kermel, Tianjin, China). Then, the PFC of mice were homogenized and sonicated. After centrifugation, the supernatant was assayed for protein content using Bradford method. Samples containing equal quantities of 1300 μg of protein were diluted with rehydration buffer to obtain (in each case) 440 μL of solution and then incubated overnight with nonlinear Immobiline Dry Strips (17 cm; pH 4–7 non-linear). The samples were then separated in the 1st dimension by isoelectric focusing (IEF). The immobilized pH gradient (IPG) strips were then incubated with equilibration buffer for 10 min followed by incubation in the same buffer with the dithiothreitol (DTT) replaced by 2.5% *w*/*v* iodoacetamide for 10 min. The strips were applied to the surface of 12% *w*/*v* SDS-PAGE gels for image analysis [43,44].

#### 3.4.2. Image Analysis of 2-DE Gels

The gels were stained with colloidal Coomassie Blue and scanned using a GS710 calibrated imaging densitometer (BioRad, Hemel Hempstead, UK). TIFF format images were analysed using Image Master TM 2D Elite software, version 4.01 (Amersham Pharmacia Biotech, Buckinghamshire, UK). 

#### 3.4.3. Identification of Proteins from 2DE Gels

Using PD Quest software (BioRad, Hemel Hempstead, UK) to find out significantly different expressed proteins among all of the groups. Protein spots of interest were excised from Colloidal Coomassie Blue-stained 2DE gels and were subjected to tryptic digestion. Peptide mass fingerprints were averaged and searched using the Group Based Prediction System database (GPS) (Applied Biosystems, Waltham, MA, USA; Life Technologies, Carlsbad, CA, USA) with MASCOT (Matrix Science, London, UK). The searching parameters were as follows: database: NCBInr; species: mice; retrieval mode: combined; enzyme: trypsin; mass error range: PMF 100 ppm, MS/MS 0.8KU. Trypsin self-degradation and contaminant peaks were removed manually [45,46].

#### 3.4.4. Bioinformatics Analysis

The FASTA sequences were extracted from database based on these interested protein identifiers, then Blast algorithm was performed against the sequence databases. The corresponding uniprot accessions were extracted from the blast result file. These protein identifiers were linked to the following databases, the Quick GO (Gene Ontology), the KEGG Pathway, and SRING interactive network analysis.

#### 3.4.5. Statistical Analysis

All the values were given as mean standard deviation. All of the results were analyzed by one-way analysis of variance (ANOVA) using SPSS19.0. A *p*-value < 0.05 indicated that there was a significant difference.

## 4. Conclusions and Expectations

The main way to study spatial radiation therapy is by using animal models, to explore the hazards, mechanisms, and drug targets of space radiation, which can help to solve the risk of irradiation in space. In this article, we demonstrated that the active compounds in AS extract had different pharmacokinetic characteristics in mouse model. Also, in our study, we observed that eight different kinds of proteins extracted from irradiated mice PFC were significantly changed after mice were orally administered AS extract, which indicated that the effective ingredients of AS could be effective through the blood–brain barrier to repair radiation damage. These data provide a new perspective on human radiation protection, whereby the application of traditional Chinese medicine can provide medical support for on-orbit flights and offer space logistical support for the moon base, also being relevant for particular civilians, such as in the prevention of electromagnetic radiation in daily life, and medical treatment or psychological recovery after a disaster. Further studies will need to focus on identifying the precise mechanisms of regulation of these proteins during the anti-radiation response and related action pathways.

## Figures and Tables

**Table 1:** Content of the active compounds in different tissues of *Acanthopanax senticosus* (AS).

Compounds	Content of the Active Compounds (%)				
	Tissue of Lesser Khingan Mountain *Acanthopanax senticosus*				
	Root	Stem	Leaf	Fruit	Average
Polysaccharides	6.05 ± 0.25	8.09 ± 0.37	4.12 ± 0.19	3.08 ± 0.13	5.33 ± 0.24
Flavones	14.25 ± 0.66	9.26 ± 0.45	4.02 ± 0.17	9.58 ± 0.50	9.28 ± 0.45
Syringin	1.44 ± 0.06	1.86 ± 0.08	0.09 ± 0.01	0.02 ± 0.001	3.40 ± 0.04
Eleutheroside E	3.17 ± 0.12	4.14 ± 0.25	0.18 ± 0.01	0.04 ± 0.002	1.88 ± 0.10

**Table 2:** Pharmacokinetics parameters of ASPS in mice’s serum.

Parameters	ASPS	
	Normal-AS-Treatment	Radiation-AS-Treatment
$t_{1/2}/h$	1.18 × 10^1^ ± 6.85 × 10^−1^	6.14 × 10^0^ ± 7.82 × 10^−1^ **
$T_{\max}/h$	2.50 × 10^−1^ ± 3.81 × 10^−1^	5.00 × 10^−1^ ± 1.44 × 10^−1^
$C_{\max}/\mathrm{ng}\cdot {\mathrm{mL}}^{-1}$	1.15 × 10^6^ ± 8.13 × 10^4^	1.01 × 10^6^ ± 1.24 × 10^5^
$\mathrm{AUC}(0\to \infty )/\mathrm{ng}\cdot h\cdot {\mathrm{mL}}^{-1}$	1.43 × 10^7^ ± 3.00 × 10^6^	8.92 × 10^6^ ± 8.81 × 10^5^ *
MRT(0→∞)/h	1.59 × 10^1^ ± 9.63 × 10^−1^	9.07 × 10^0^ ± 1.95 × 10^−1^ **
$\mathrm{Vd}/L\cdot g^{-1}$	9.83 × 10^−4^ ± 1.76 × 10^−4^	8.19 × 10^−4^ ± 8.33 × 10^−5^
$\mathrm{CL}/L\cdot h^{-1}\cdot g^{-1}$	5.76 × 10^−5^ ± 6.12 × 10^−6^	9.25 × 10^−5^ ± 8.50 × 10^−6^ *

* *p* < 0.05; ** *p* < 0.01 (compared with normal-AS-treatment group).

**Table 3:** Pharmacokinetics parameters of Syringin and Eleutheroside E in mice’s serum.

Parameters	Syringin		Eleutheroside E	
	Normal-AS-Treatment	Radiation-AS-Treatment	Normal-AS-Treatment	Radiation-AS-Treatment
$t_{1/2}/h$	6.47 × 10^0^ ± 6.61 × 10^−1^	9.72 × 10^0^ ± 3.81 × 10^−1^ ***	1.72 × 10^1^ ± 7.22 × 10^−1^	2.51 × 10^1^ ± 5.56 × 10^0^ **
$T_{\max}/h$	5.00 × 10^−1^ ± 1.83 × 10^−1^	5.00 × 10^−1^ ± 2.37 × 10^−1^	5.00 × 10^−1^ ± 1.92 × 10^−1^	5.00 × 10^−1^ ± 3.21 × 10^−1^
$C_{\max}/\mathrm{ng}\cdot {\mathrm{mL}}^{-1}$	1.70 × 10^2^ ± 7.41 × 10^0^	2.12 × 10^2^ ± 1.97 × 10^1^ ***	2.07 × 10^3^ ± 6.61 × 10^2^	2.11 × 10^3^ ± 5.93 × 10^2^
$\mathrm{AUC}(0\to \infty )/\mathrm{ng}\cdot h\cdot {\mathrm{mL}}^{-1}$	2.83 × 10^2^ ± 3.23 × 10^1^	5.34 × 10^2^ ± 6.54 × 10^−1^	9.93 × 10^3^ ± 1.81 × 10^3^	1.20 × 10^4^ ± 3.63 × 10^3^ *
MRT(0→∞)/h	4.58 × 10^0^ ± 6.12 × 10^−1^	5.95 × 10^0^ ± 7.30 × 10^−1^	1.48 × 10^1^ ± 5.97 × 10^−1^	2.11 × 10^1^ ± 7.01 × 10^−1^ **
$\mathrm{Vd}/L\cdot g^{-1}$	2.72 × 10^1^ ± 8.73 × 10^−1^	2.17 × 10^1^ ± 9.19 × 10^−1^ **	2.06 × 10^0^ ± 8.87 × 10^−1^	2.49 × 10^0^ ± 4.49 × 10^−1^ *
$\mathrm{CL}/L\cdot h^{-1}\cdot g^{-1}$	2.91 × 10^0^ ± 5.65 × 10^−1^	1.54 × 10^0^ ± 1.86 × 10^−1^	8.30 × 10^−2^ ± 7.32 × 10^−3^	6.89 × 10^−2^ ± 7.17 × 10^−3^ **

* *p* < 0.05; ** *p* < 0.01; *** *p* < 0.001 (compared with normal-AS-treatment group).

**Table 4:** Basic information of differential proteins (partial).

Sample	Protein Name	Accession No	Homology Model	Score	Identity	Gene Name
3307↑	Tubulin α-1A chain	gi|55977479	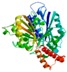	779	99.54	*Tuba1b*
3315↓	Tubulin β-2A chain	gi|81885934	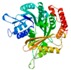	1020	100	*Tubb2a*
4422↑	γ-actin	gi|809561	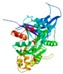	912	100	*Actg1*
8116↓	14-3-3 protein ζ (Homo sapiens)	gi|148676868	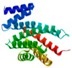	644	100	*Ywhaz*
7119↓	14-3-3 protein ξ (Homosapiens)	gi|26344914	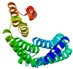	516	99.61	*Ywhae*
0314↑	Heat shock protein HSP 90-β	gi|341941065	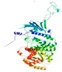	685	100	*Hsp90ab1*
2814↓	dihydropyrimidinase-related protein 2	gi|568986628	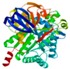	1150	98.6	*Dpysl2*
3311↓	enolase 2, γ neuronal	gi|148667340	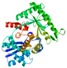	595	100	*Eno2*

↑: The protein’s gray scale was significantly higher in AS treatment group than the model set; ↓: The protein’s gray scale was significantly lower in AS treatment group than model set.

**Table 5:** Kyoto Encyclopedia of Genes and Genomes (KEGG) pathways of differential proteins (partial).

Sample	Protein Name	KEGG Pathways
3307	α-tubulin	mmu04540, Gap junction|mmu04210, Apoptosis|mmu04145, Phagosome
3155	β-tubulin	mmu04540, Gap junction|mmu04145, Phagosome
4422	γ-actin	mmu04530, Tight junction|mmu04670, Leukocyte transendothelial migration|mmu05414, Dilated cardiomyopathy|mmu04015, Rap1 signaling pathway|mmu05416, Viral myocarditis|mmu05410, Hypertrophic cardiomyopathy (HCM)|mmu04510, Focal adhesion|mmu05205, Proteoglycans in cancer|mmu04520, Adherens junction|mmu04611, Platelet activation|mmu05100, Bacterial invasion of epithelial cells|mmu04210, Apoptosis|mmu04145, Phagosome|mmu05412, Arrhythmogenic right ventricular cardiomyopathy (ARVC)|mmu05132, Salmonella infection|mmu04390, Hippo signaling pathway|mmu04810, Regulation of actin cytoskeleton|mmu04921, Oxytocin signaling pathway|mmu04919, Thyroid hormone signaling pathway|mmu05164, Influenza A
8116	14-3-3ζ	mmu05203, Viral carcinogenesis|mmu05161, Hepatitis B|mmu04114, Oocyte meiosis|mmu05169, Epstein-Barr virus infection|mmu04110, Cell cycle|mmu04151, PI3K-Akt signaling pathway|mmu04390, Hippo signaling pathway
7119	14-3-3ε	mmu05203, Viral carcinogenesis|mmu04722, Neurotrophin signaling pathway|mmu04114, Oocyte meiosis|mmu05169, Epstein-Barr virus infection|mmu04110, Cell cycle|mmu04151, PI3K-Akt signaling pathway|mmu04390, Hippo signaling pathway
0314	HSP 90-β	mmu04915, Estrogen signaling pathway|mmu04914, Progesterone-mediated oocyte maturation|mmu04621, NOD-like receptor signaling pathway|mmu04612, Antigen processing and presentation|mmu05215, Prostate cancer|mmu04141, Protein processing in endoplasmic reticulum|mmu04151, PI3K-Akt signaling pathway|mmu05200, Pathways in cancer
2814	CRMP2	mmu04360, Axon guidance
3311	ENO2	mmu01100, Metabolic pathways|mmu01200, Carbon metabolism|mmu04066, HIF-1 signaling pathway|mmu01230, Biosynthesis of amino acids|mmu00010, Glycolysis/Gluconeogenesis|mmu03018, RNA degradation
